# Sepsis in cancer patients residing in Zimbabwe: spectrum of bacterial and fungal aetiologies and their antimicrobial susceptibility patterns

**DOI:** 10.1186/s12879-020-4886-2

**Published:** 2020-02-21

**Authors:** Frank Chinowaita, Wendy Chaka, Tinashe K. Nyazika, Tendai C. Maboreke, Emmanuel Tizauone, Prichard Mapondera, Inam Chitsike, Andrew Z. Cakana, Rooyen T. Mavenyengwa

**Affiliations:** 1 Department of Medical Microbiology, College of Health Sciences, University of Zimbabwe, Harare, Zimbabwe; 2 Premier Services Medical Investments, Department of Microbiology, Harare, Zimbabwe; 3 Department of Pathology (Microbiology), Midlands State University, Gweru, Zimbabwe; 4 Malawi-Liverpool-Welcome Trust Clinical Research Programme, College of Medicine, University of Malawi, Blantyre, Malawi; 5 Department of Clinical Sciences, Liverpool School of Tropical Medicine, Liverpool, UK; 6 Department of Medical Microbiology, Radboud University Medical Centre, Nijmegen, The Netherlands; 7 Department of Haematology, College of Health Sciences, University of Zimbabwe, Harare, Zimbabwe; 8 African Society Laboratory Medicine, Addis Ababa, Ethiopia; 9 Department of Paediatrics and Child Health, College of Health Sciences, University of Zimbabwe, Harare, Zimbabwe

**Keywords:** Sepsis, Cancer, Aetiology, Antimicrobial resistance, ESBL

## Abstract

**Background:**

Cancer and sepsis comorbidity is a major public health problem in most parts of the world including Zimbabwe. The microbial aetiologies of sepsis and their antibiograms vary with time and locations. Knowledge on local microbial aetiologies of sepsis and their susceptibility patterns is critical in guiding empirical antimicrobial treatment choices.

**Methods:**

This was a descriptive cross-sectional study which determined the microbial aetiologies of sepsis from blood cultures of paediatric and adult cancer patients obtained between July 2016 and June 2017. The TDR-X120 blood culture system and TDR 300B auto identification machine were used for incubation of blood culture bottles and identification plus antimicrobial susceptibility testing, respectively.

**Results:**

A total of 142 participants were enrolled; 50 (35.2%) had positive blood cultures, with 56.0% Gram positive, 42.0% Gram-negative bacteria and 2.0% yeast isolated. Common species isolated included coagulase negative *Staphylococcus* spp. (CoNS) (22.0%), *E. coli* (16.0%), *K. pneumoniae* (14.0%), *E. faecalis* (14.0%) and *S. aureus* (8.0%). Gram-negative isolates exhibited high resistance to gentamicin (61.9%) and ceftriaxone (71.4%) which are the empiric antimicrobial agents used in our setting. Amikacin and meropenem showed 85.7 and 95.2% activity respectively against all Gram-negative isolates, whilst vancomycin and linezolid were effective against 96.2 and 100.0% of all Gram-positive isolates respectively. We isolated 10 (66.7%) extended spectrum β-lactamase (ESBL) amongst the *E. coli* and *K. pneumoniae* isolates. Ten (66.7%) of the *Staphylococcus* spp. were methicillin resistant.

**Conclusions:**

CoNS, *E. coli*, *K. pneumoniae*, *E. faecalis* and *S. aureus* were the major microbial drivers of sepsis amongst cancer patients in Zimbabwe. Most isolates were found to be resistant to commonly used empirical antibiotics, with isolates exhibiting high levels of ESBL and methicillin resistance carriage. A nationwide survey on microbial aetiologies of sepsis and their susceptibility patterns would assist in the guidance of effective sepsis empiric antimicrobial treatment among patients with cancer.

## Background

Despite the major advances in the care of patients with cancer over the past few decades and the resultant improvement in survival, complications during the course of disease arise that are associated with significant morbidity and mortality [1]. Cancer is one of the leading risk factors of developing sepsis, with cancer patients having a 10-fold relative risk compared to non-cancer patients [2]. In addition to being a leading cause of hospitalisation in this population, sepsis represents a common pathway of mortality among cancer patients [3]. The comorbidity of sepsis and cancer poses serious complications with very poor prognosis with a case fatality ratio of greater than 50% in the Americas [4]. Sepsis as a syndrome can result from healthcare–associated or community–acquired infection by organisms and these organisms can develop resistance to commonly prescribed antimicrobial agents [5]. Without proper determination of antimicrobial susceptibility patterns of these organisms, treatment may prove to be difficult, leading to other complications like organ failure, shock and death [6].

Among cancer patients with sepsis the organisms commonly isolated are bacterial or fungal pathogens, with the predominant pathogens being *Staphylococcus aureus*, *Pseudomonas* species, *Escherichia coli*, and *Candida* species [1, 7]. Laboratory investigations in sepsis include measurement of inflammatory markers, organ function tests and identification of infectious source through blood culture plus any culture specimens to identify source of infection [5, 8]. In Zimbabwe, sepsis diagnosis is primarily clinically based and confirmation of infection with blood cultures is not always adhered to particularly in the public health institutions.

According to guidelines in Zimbabwe, sepsis is empirically treated with gentamicin and either benzylpenicillin or cloxacillin with ceftriaxone and chloramphenicol being used as empiric antibiotics when involvement with the central nervous system is suspected [9]. Evidence from literature demonstrates variations in aetiological agents of sepsis in different geographical settings, thus microbial and antimicrobial profiling should be country/region specific [6, 7, 10, 11]. With the rise of antimicrobial resistance among clinical isolates, it is imperative to profile the causative pathogens of sepsis and their antimicrobial patterns. This could aid in reducing patient hospital costs, sepsis related complications and deaths.

To date, the burden of sepsis in cancer patients and or their causative pathogens remain sparse in Zimbabwe and Africa at large, despite the growing burden of cancer. Thus, this study aimed to ascertain the microbial agents of sepsis and their antimicrobial susceptibility patterns among hospitalised paediatric and adult patients with cancer in Zimbabwe.

## Methods

### Study design and study population

Between July 2016 and June 2017, we performed a prospective descriptive cross-sectional study among hospitalised paediatric and adult haematology/oncology patients at a single centre, Parirenyatwa Group of Hospitals. It is the biggest and major referral centre for patients with cancer in Zimbabwe and is located in the capital city, Harare. The target population were paediatric patients at least 1-year of age and adult patients who had a diagnosis of cancer, presenting with signs of sepsis. The participants included had to have the following; suspected infection with at least fever (< 36 °C or > 38 °C), heart rate (> 90 bpm) and white cell count (< 4.0 × 10^9^ or > 12.0 × 10^9^/L). Clinical assessment of sepsis was done using the quick Sequential Organ Failure (qSOFA) score which includes (1) respirations > 22 breaths/minute, (2) altered mentation, (3) systolic blood pressure < 100 mmHg, with two or more considered ‘high’ risk [12]. The qSOFA score ranges from 0 to 3, with each criterion being worth one point. When respiration rate, altered mentation, or systolic blood pressure data was not available, the corresponding criterion was set to be worth zero point. For the 48% of patients (68/142) for whom clinical data was complete, the qSOFA score [12], including (1) creatinine > 110 μMol/L, (2) platelets < 150 × 103/μL, and (3) total bilirubin > 20 μMol/L was also calculated.

### Sample collection and analysis

At least two peripheral vein blood samples were consecutively drawn aseptically for blood cultures from paediatrics (3 ml each) and adult (8 ml each) per participant. The TDR Resin Aerobic or TDR Resin Peds (Hunan Changsha Tiandiren Bio-Tech Co., Ltd., Changsha, China) blood culture bottles, which support growth of both aerobic bacteria and mycotic yeasts, were used for sample collection from participants. The collected blood culture samples were processed and cultured using standard microbiology hospital protocols. Briefly, TDR Resin Aerobic or TDR Resin Peds (Hunan Changsha Tiandiren Bio-Tech Co., Ltd., Changsha, China) blood culture bottles, from the participants were incubated at 37 °C in an automated microbial detection blood culture system TDR-X120 (Hunan Changsha Tiandiren Bio-Tech Co., Ltd., Changsha, China). Blood cultures read as positive by the analyser were immediately retrieved, Gram stained and sub-cultured on Blood agar, MacConkey agar, Chocolate agar and Sabouraud dextrose agar supplemented with chloramphenicol (0.5 g/l) (all Mast Group Ltd., Merseyside, UK) plates for 48-h. The blood culture system has an incubation period of up to 5-days, after which it reports a blood culture specimen as negative for growth. All negative blood cultures, as read by the machine, were also Gram stained and sub-cultured similarly as the positive ones to confirm the negative result. We only considered a patient to be infected, when at least two of the blood cultures had been positive. A single positive blood culture result was interpreted as possible contamination.

### Identification and antimicrobial susceptibility testing of isolates

Isolates grown from culture plates were initially identified as lactose fermenting coliform, non-lactose fermenting coliform, oxidase positive (non-fermenter) Gram-negative rods, *Staphylococcus*, *Streptococcus* species or yeasts based on colony morphology. These were further speciated by means of various biochemical tests and antimicrobial susceptibility test (AST) using standard methods on the Mindray TDR 300B (Hunan Changsha Tiandiren Bio-Tech Co., Ltd., Changsha, China) following the manufacturer’s manual. Probabilities were calculated from these results using the Bifido-Matrix method to identify the most possible organism. Antimicrobial susceptibility testing plates were read on the TDR 300B based on turbidity and interpretations were made using breakpoints stipulated in the Clinical and Laboratory Standards Institute (CLSI) 2017 guidelines [13]. Isolates found to be multidrug resistant were tested for Extended Spectrum β-Lactamase production, methicillin resistance and carbapenemase production as described in the CLSI standard [13]. *Pseudomonas aeruginosa* ATCC® 27853, *E. coli* ATCC® 25922 and *S. aureus* ATCC® 25923 strains were used for quality control (QC) during identification and AST on the Mindray TDR 300B machine.

### Statistical analysis

Characteristics of the study participants were analysed using descriptive statistics with results expressed as frequencies and percentages. Aetiological profiles were described for the overall sample using percentages and their distribution by cancer types. All data analysis was performed using Stata software *v*13 (StataCorp). Observations with missing values were coded as missing and reported as such.

## Results

### Demographic characteristics of the population

A total of 142 consecutive hospitalised cancer participants with clinical diagnosis of sepsis were recruited into the study, with females 76 (53.5%) and paediatric patients 86 (60.6%) being the majority. The age ranged between 1 and 85 years, with an overall median age 10 (interquartile range [IQR]: 5–24) years and a median in-patient hospital stay of 7 (IQR: 4–15) days before diagnosis of sepsis was suspected. One hundred and ten (77.5%) participants had haematological neoplasms which comprised mostly of leukaemias and lymphomas whilst 32 (22.5%) had solid tumours such as Wilms tumour, rhabdomyosarcoma and hepatocellular carcinoma. Neutropenia, one of the major sepsis risk factors, was assessed from the patients’ absolute neutrophil counts. The absolute neutrophil count of patients on blood culture sample collection ranged between 20 and 102,700 cells/μl. Neutropenia (< 1000 cells/μl) as previously defined in other studies [7, 14] was observed in 43 (39.1%) of the participants with haematological neoplasm and one participant with a solid tumour giving a total of 44 (31.0%) neutropenic patients. There was a strong association between having a haematological neoplasm and being neutropenic (Odds Ratio, 19.9; 95% CI 3.0–829.2; p- < 0.001). Participants’ demographic characteristics are summarised in Table 1.

**Table 1:** Study population demographic characteristics

Characteristic	Total	Haematological neoplasm	Solid tumour
Female *n* (%)	76 (53.5)	58 (76.3)	18 (23.7)
Age median (IQR) years	–	15 (4–34)	6 (5–10)
Paediatric (oncology ward) *n* (%)	86	55 (64.0)	31 (36.0)
Adults (oncology ward) *n* (%)	1	–	1 (100.0)
Adult (haematology ward) *n* (%)	55	55 (100.0)	–
Hospital stay median (IQR) days	7.0 (4–15)	7.0 (4–15)	7.5 (3–13)
Neutropenia *n* (%)	44 (31.0)	42 (39.1)	1 (3.1)

*n,* number

### Blood cultures and pathogens isolated

Of the 142 participants, fifty (35.2%) had positive blood cultures. Thirty-nine of the 110 patients with haematological malignancies had positive blood cultures with a positive isolation rate of 35.5% contributing 78% of the total number of isolates. Gram-positive bacterial pathogens were the predominant 28 (56.0%) of the causative agents of sepsis in this population with coagulase negative *Staphylococcus* spp. (CoNS) being the majority contributing 22.0% of the pathogens isolated. *E. coli* was the second most abundant 8 (16.0%) species isolated. *Candida albicans* was the only fungal pathogen isolated from one participant with sepsis in this study. Table 2 summarises our findings.

**Table 2:** Distribution of sepsis causing pathogens in participants with cancer

Causative pathogen	Number of isolates (*n*)				Total (%)
	Haematological neoplasm		Solid tumour		
	Children	Adults	Children	adults	
Gram-negative bacteria (*n* = 21)					
*Escherichia coli*	4	2	2	–	8 (16.0)
*Klebsiella pneumoniae*	2	3	2	–	7 (14.0)
*Enterobacter intermedium*	1	–	–		1 (2.0)
*Serratia odorifera*	–	1	–	–	1 (2.0)
*Acinetobacter species*	1	–	–	–	1 (2.0)
*Pseudomonas aeruginosa*	1	–	–	–	1 (2.0)
*Salmonella enteritidis*	–	1	–	–	1 (2.0)
*Hafnia alvei*	–	–	1	–	1 (2.0)
Gram positive bacteria (*n* = 28)					
CoNS	3	6	2	–	11 (22.0)
*Staphylococcus aureus*	2	1	–	1	4 (8.0)
*Enterococcus faecalis*	1	5	1	–	7 (14.0)
*Enterococcus gallinarum*	2	–	–	–	2 (4.0)
*Enterococcus faecium*	1	–	–	–	1 (2.0)
*Streptococcus* species	0	1	2	–	3 (6.0)
Fungi (*n* = 1)					
*Candida albicans*	1	–	–	–	1 (2.0)

**n,** number

### Exposure to antimicrobials and antimicrobial susceptibility profiles

One hundred and twenty-nine 129(90.8%) of our participants were exposed to at least one antimicrobial agent at least 48-h prior to blood culture collection. The most commonly prescribed antibiotic was ceftriaxone 100/129 (77.5%) followed by gentamicin 75/129 (58.1%) and ciprofloxacin 33 (25.6%). Twenty-seven (20.9%) participants were on fluconazole therapy. At least 3 antibiotics had been administered to 58.0% of the participants prior to blood culture collection.

After performing AST on the isolates, *Staphylococci* spp. had the highest resistance to penicillin 14 (93.7%), with methicillin resistance observed in 10 (66.7%) of the *Staphylococci* isolates. Based on CLSI 2017 guideline, the same results can be applied to cloxacillin, augmentin and cefazolin. However, all the isolates were fully susceptible to vancomycin and linezolid.

Among the Gram-negative bacterial isolates, antibiotics such as levofloxacin (52.4%), cefepime (61.9%), cefoxitin (66.7%), piperacillin-tazobactam (71.9%), amikacin (85.7%) and meropenem (95.2%) exhibited moderate to high potency against all Gram-negative isolates. Ampicillin and trimethoprim-sulfamethoxazole were least effective with only 4.8% of the isolates being sensitive. High level of resistance was observed among *K. pneumoniae* followed by *E. coli* isolates. Among *K. pneumoniae* isolates, resistance was observed in ampicillin (100%), trimethoprim-sulfamethoxazole (85.7%) and third generation cephalosporins (71.4%) respectively. Resistance to gentamicin, one of the first line empiric antimicrobial in our setting, was 57.1% among *K. pneumoniae* isolates. Against third generation cephalosporins that is ceftriaxone, an empiric antimicrobial in the local Essential Medicines List and Standard Treatment Guidelines for Zimbabwe (EDLIZ), and ceftazidime, resistance was observed in 71.4% of these isolates. However, isolates were fully sensitive to amikacin and meropenem and moderately sensitive to cefoxitin (85.7%). *E. coli* isolates were also fully susceptible to meropenem and amikacin while 75.0% of the isolates were resistant to ceftriaxone, ceftazidime, gentamicin, ciprofloxacin and levofloxacin (see Table 4). Trimethoprim-sulfamethoxazole and ampicillin displayed the least activity against *E. coli* isolates with sensitivities of 0.0 and 12.5% respectively. Other isolates were few to make inferences as they were only a single isolate of each species. These included *Serratia odorifera*, *Acinetobacter* species, *Salmonella enteritidis*, *Enterobacter intermedium* and *Hafnia alvei*. Of note, the *S. odorifera* was only sensitive to levofloxacin and resistant to meropenem and ertapenem. Overall, the proportions of isolates resistant to empiric antimicrobial agents in Zimbabwe (gentamicin and ceftriaxone) among *Enterobacteria* species were 61.9% and 71.4% respectively.

When we investigated the *Enterococcus* species, the isolates were fully susceptible to linezolid and vancomycin, while they showed high resistance to tetracycline 2 (20.0%) and ciprofloxacin 4 (40.0%). *E. gallinarum* was resistant to the majority of drugs with the two isolates being sensitive to vancomycin and linezolid. *Streptococcus* species on the other hand were resistant (66.7%) to tetracycline, ampicillin and penicillin. One *Streptococcus* species, which was identified as *Streptococcus bovis,* showed resistance to vancomycin. Overall, 69.2% isolates of *Enterococcus* and *Streptococcus* species were susceptible to the empiric antimicrobial agents, high dose gentamicin and penicillin.

Finally, a single isolate of *C. albicans* was the only fungal pathogen isolated from the blood cultures. It proved to be resistant to terbinafine, itraconazole and fluconazole. However, the isolate was sensitive to other antifungals such as micafungin, caspofungin, voriconazole, ketoconazole, miconazole, amphotericin B and flucytosine. Tables 3, 4 and 5 summarises the antimicrobial susceptibility patterns of all the isolates.

**Table 3:** Distribution of drug susceptible *Staphylococcus* species

Bacterial species isolates	N	VA	LIN	ERY	CD	TET	MINO	RIF	GM	CIP	PEN	COT
*S. aureus*	4	4	4	2	3	3	3	3	3	2	0	2
*CoNS*	11	11	11	7	7	5	9	8	6	5	1	6
Total sensitive *n* (%)	**15(100)**	**15(100)**	**15(100)**	**9(60.0)**	**10(66.7)**	**8(53.3)**	**12(80.0)**	**11(73.3)**	**9(60.0)**	**7(46.7)**	**1(6.7)**	**8(53.3)**

Notes: *VA* Vancomycin, *LIN* Linezolid, *ER*Y Erythromycin, *CD* Clindamycin, *TET* Tetracycline, *MINO* Minocycline, *RIF* Rifampicin, *CHL* Chloramphenicol, *CIP* Ciprofloxacin, *GM* Gentamicin, *PEN* Penicillin, *COT* Trimethoprim-sulfamethoxazole, *N* Number; (0), zero susceptible isolates

**Table 4:** Distribution of antimicrobial susceptibility patterns for gram negative isolates

Bacterial isolates	N	AMP	PTZ	CXM	CRO	CAZ	CEF	FOX	GM	AK	CIP	LEV	MEM	COT
*E. coli*	8	1	7	2	2	2	4	7	2	8	2	2	8	0
*K. pneumoniae*	7	0	4	2	2	2	4	6	3	7	3	4	7	1
*E. intermedium*	1	0	1	0	0	0	1	0	1	0	1	1	1	0
*S. odorifera*	1	0	0	0	0	0	0	0	0	0	0	1	0	0
*S. enteritidis*	1	0	1	0	1	1	1	0	0	0	1	1	1	0
*Acinetobacter sp.*	1	0	0	0	1	1	1	0	0	1	0	0	1	0
*P. aeruginosa*	1	–	1	–	0	1	1	–	1	1	1	1	1	0
*H. alvei*	1	0	1	0	0	0	1	1	1	1	1	1	1	0
Total *n* (%)	**21 (100)**	**1(4.8)**	**15(71.4)**	**4(19.0)**	**6(28.6)**	**7(33.3)**	**13(61.9)**	**14(66.7)**	**8(38.1)**	**18(85.7)**	**9(42.9)**	**11(52.4)**	**20(95.2)**	**1(4.8)**

Notes: *AMP* Ampicillin, *PTZ* Piperacillin-tazobactam, *CRO* Ceftriaxone, *CAZ* Ceftazidime, *CXM* Cefuroxime, *CEF* Cefepime, *FOX* Cefoxitin, *GM* Gentamicin, *AK* Amikacin, *CIP* ciprofloxacin, *LEV* Levofloxacin, *COT* Trimethoprim-sulfamethoxazole, MEM Meropenem; *N* Number; (−), not tested, (0), zero sensitive isolates

**Table 5:** Antimicrobial susceptibility patterns for *Streptococcus* and *Enterococcus* species

Bacterial isolates	N	AMP	PEN	VA	LINE	FOSF	GM	TET	CIP	LEV	GATI
*E. faecalis*	7	7	7	7	7	7	6	2	4	4	4
*E. gallinarum*	2	0	0	2	2	1	0	0	0	0	0
*E. faecium*	1	1	1	1	1	1	1	0	0	1	1
*Streptococcus sp.*	3	1	1	2	3	2	2	1	2	2	2
Total *N* (%)	**13(100)**	**9(69.2)**	**9(69.2)**	**12(92.3)**	**13(100)**	**11(84.6)**	**9(69.2)**	**3(23.1)**	**6(46.2)**	**7(53.8)**	**7(53.8)**

Notes: *AMP* Ampicillin, *PEN* Penicillin, *VA* Vancomycin, *LINE* Linezolid, *FOSF* Fosfomycin, *GM* Gentamicin, *TET* Tetracycline, *LEV* Levofloxacin, *CIP* Ciprofloxacin, *GATI* Gtifloxacin, *N* Number, (0), zero sensitive isolates

### Incidence of ESBL production among *E. coli* and *K. pneumoniae* isolates

Fifteen isolates of both *E. coli* and *K. pneumoniae* obtained in this study were screened for *ESBL* enzyme production and 10 (66.7%) were phenotypically confirmed to be *ESBL* producers. *E. coli* isolates were the main *ESBL* producers with 6/8 (75.0%) of the isolates being positive. Four (57.1%) of the total *K. pneumoniae* isolates were also confirmed *ESBL* producers.

## Discussion

Sepsis is a serious life-threatening condition that commonly manifests itself in the cancer patients. Although there are studies that have been conducted in Africa on cancer patients presenting with sepsis [10, 15], limited data regarding the profiles of the organisms implicated and antibiotic susceptibility data exist. In this study we report the isolation rate of bacterial and fungal pathogen from blood cultures of cancer patients (both adults and paediatric) presenting with sepsis, as well as the antimicrobial profiles of commonly used antibiotics in our setting. We also demonstrate that there is a high level of resistance among pathogens causing sepsis in our setting.

Patients with haematological malignancies were the majority (77.5%) and this could be due to neutropenia secondary to chemotherapy which further exposes them to infections. The overall proportion of the patients who were neutropenic was 31.0% which is similar to the 30.0% reported in the USA [7]. Patients with haematological malignancies showed a significantly higher proportion of neutropenia compared to those with solid cancers, a finding similar to the Chinese and European populations [14, 16].

The majority (90.8%) of the study participants were on at least one antimicrobial agent at least 48 h prior to blood culture collection and this was as a consequence of their immunosuppression being caused by the cancer. However, it was also observed that 82 (57.7%) were on a cocktail of 3 to 6 broad spectrum antimicrobial agents contrary to the standard empirical treatment of sepsis stipulated in the local EDLIZ [9]. Ceftriaxone and gentamicin were the major empirical antibiotics used despite the recommendations that ceftriaxone should only be used as second line [9]. Use of ceftriaxone and other antimicrobials as first line empirical antimicrobial treatment could be due to limited knowledge on the implications such as antimicrobial resistance and presumed resistance to prescribed empiric treatment.

Our microbial pathogen isolation rate was 35.2% which is slightly higher than the average of 20 and 30% range in most studies [2, 4, 8]. Other studies from high income countries have, on the contrary, reported lower prevalence of sepsis among patients with cancer including studies in Oman (5.0%) and Europe (17%) [14, 17]. Among the isolates identified, Gram-positive to Gram-negative percentage ratio was 57:43 which was comparable with the median ratio of 60:40 (range 85:15 to 26:76) obtained in Europe [18, 19]. This reflects a similarity in the distribution of organisms despite geographical differences although minor difference can be encountered, like a study in Sudan where the ratio was 83%:17% [10]. Most of the isolates (78.0%) came from patients with haematological malignancies, a finding comparable to other earlier studies [4, 14]. The major aetiological agents of sepsis obtained from patients with haematological cancers were CoNS, *E. coli*, *E. faecalis* and *K. pneumoniae*. Similarly, other studies from Europe have reported the same organisms as the causative agents of sepsis but with some minor variations in proportions [14, 18]. Most studies had not stratified aetiological agents with cancer type but a study in Europe with the same stratification showed similar aetiological agents between the two major cancer groups [14].

Amikacin and meropenem were the most potent drugs against Gram-negative isolates with more than 80.0% of the isolates being sensitive, similar to findings from a study in the USA [7]. Conversely, more than 60.0% of the isolates were resistant to third generation cephalosporins, in contrast with the USA and an earlier study in Zimbabwe where 80–100% were sensitive [7, 20]. This difference could be due to the wide availability and uncontrolled use of ceftriaxone as first line treatment, as was found in this study. As also shown in this and other studies [18, 21–23], the increase in the emergence of ESBL producing isolates has also led to this high level of resistance to the third generation cephalosporins. Gentamicin, the most commonly used empirical aminoglycoside, also had a low activity against these Gram-negative isolates as > 60.0% of the isolates were resistant. Resistance to third generation cephalosporins and gentamicin has been reported in earlier studies to be rising in low-income countries [22, 24]. Such resistance to the empiric antimicrobial agents poses a challenge in the management of sepsis among this population as it limits treatment options hence the need to review empirical treatment options. Cefoxitin and piperacillin-tazobactum were effective against 66.7 and 71.4% of all the Gram-negative isolates. However, more than 90.0% of the isolates were resistant to trimethoprim-sulfamethoxazole and ampicillin, a finding similar to most studies worldwide [16, 20, 24]. The resistance to trimethoprim-sulfamethoxazole has been attributed to overuse of the drug as prophylaxis against *Pneumocystis jirovecii* pneumonia in HIV endemic regions such as Zimbabwe. Notably, there was one *S. odorifera* isolate that was resistant to meropenem and ertapenem. This is surprising as carbapenem resistance *Enterobacteriaceae* has not been reported before in Zimbabwe. However, the isolate was not confirmed with polymerase chain reaction for carbapenemase resistance gene carriage. Nevertheless, this could be a possible emergence of carbapenemase resistance since carbapenems are being employed routinely for management of patients in the institution under study.

Expectedly, due to their limited use locally, minocycline, chloramphenicol, linezolid and vancomycin showed to be effective against more than 80.0% of the *Staphylococcus* isolates. A moderately high activity was displayed by gentamicin, clindamycin and erythromycin. These results were partly in agreement with findings from Ghana and India [24, 25]. Conversely, there was high rate of methicillin resistance which impliedly apply to cloxacillin, one of the EDLIZ prescribed empiric antimicrobial agent. The low activity observed in penicillin was previously reported in Ghana, India and Zimbabwe [20, 24, 25]. *Enterococcus* and *Streptococcus* species in our study were highly sensitive to fosfomycin, vancomycin and linezolid with the latter being the most effective (isolates were 100.0% sensitive) antibiotic. Contrary to findings in India where they found 50% of *Enterococcus* species to be resistant to vancomycin, all our isolates were sensitive to vancomycin [26]. These isolates also displayed a moderate sensitivity to gentamicin, ampicillin and penicillin. Surprisingly, one isolate of *Streptococcus bovis* was resistant to vancomycin, a finding that has not been reported before in Zimbabwe. However, vancomycin resistance amongst *Streptococcus bovis* has been reported before in some parts of the world [27].

Some isolates phenotypically showed multidrug resistance capabilities. Our methicillin resistance carriage was comparable to USA isolates where MRSA was 50.0% in our current study versus 41.0% in USA while that of methicillin resistant CoNS was 75.0% versus 72.0% respectively [7]. In Ghana, a low proportion of MRSA (5.8%) was reported in contrast to our findings [24]. This high-level methicillin resistance limits the choices of antimicrobial treatment since it also implies that these isolates will also be clinically resistant to most if not all commonly used beta-lactam antibiotics. We also found a high proportion of ESBL producers among *E. coli* and *K. pneumoniae* isolates and this was in agreement with some studies around the world [18, 21, 22, 28]. However, of note was a higher proportion of ESBL producing *E. coli* (75.0%) than *K. pneumoniae* (57.1%), a different finding from most reports in other parts of the world where ESBL production is predominantly found in *K. pneumoniae* isolates [21, 22].

## Conclusion

In summary, sepsis remains a leading cause of morbidity and mortality among patients with cancer; with the major aetiological agents being CoNS, *E. coli*, *K. pneumoniae*, *E. faecalis* and *S. aureus*. Similar aetiological pathogens were present in both haematological and solid cancers in the Zimbabwean population. Most of the microbial aetiological agents of sepsis showed high levels of resistance to commonly used antimicrobial drugs as well as to those prescribed as local empirical treatment. Resistance to gentamicin, penicillin and third generation cephalosporins is a major cause for concern as these are the major empirical antibiotics in resource limited settings. Apart from vancomycin, linezolid was shown to be another better option to be considered in the treatment of serious and non-responsive Gram-positive infections while amikacin and meropenem can also be considered in Gram-negative infections. The emergence of multidrug resistance mechanisms like ESBL, carbapenemase carriage and methicillin resistance among isolates is disturbing and this demonstrates the need for active surveillance to reduce their transmission with a goal to mitigate mortality and morbidity among patients.

## Data Availability

Data for this study have been included within the document. For any further information that might be required, the corresponding author is willing to provide the information.

## Abbreviations

AST: Antimicrobial susceptibility testing
ATCC: American type culture collection
CLSI: Clinical and laboratory standard institute
CoNS: Coagulase negative *Staphylococcus*
EDLIZ: Essential medicines list and standard treatment guidelines for Zimbabwe
ESBL: Extended spectrum beta lactamase
IQR: Interquartile range
MRSA: Methicillin resistant *Staphylococcus aureus*
qSOFA: Quick sequential organ failure assessment
